# Zombie cheminformatics: extraction and conversion of Wiswesser Line Notation (WLN) from chemical documents

**DOI:** 10.1186/s13321-024-00831-2

**Published:** 2024-04-15

**Authors:** Michael Blakey, Samantha Pearman-Kanza, Jeremy G. Frey

**Affiliations:** Department of Chemistry, University of Southampton, University Road, Southampton, Hampshire SO17 1BJ UK

**Keywords:** WLN, Chemical line notation, SMILES, Chemical compounds, Text parsing

## Abstract

**Purpose:**

Wiswesser Line Notation (WLN) is a old line notation for encoding chemical compounds for storage and processing by computers. Whilst the notation itself has long since been surpassed by SMILES and InChI, distribution of WLN during its active years was extensive. In the context of modernising chemical data, we present a comprehensive WLN parser developed using the OpenBabel toolkit, capable of translating WLN strings into various formats supported by the library. Furthermore, we have devised a specialised Finite State Machine l, constructed from the rules of WLN, enabling the recognition and extraction of chemical strings out of large bodies of text. Available open-access WLN data with corresponding SMILES or InChI notation is rare, however ChEMBL, ChemSpider and PubChem all contain WLN records which were used for conversion scoring. Our investigation revealed a notable proportion of inaccuracies within the database entries, and we have taken steps to rectify these errors whenever feasible.

**Scientific contribution:**

Tools for both the extraction and conversion of WLN from chemical documents have been successfully developed. Both the Deterministic Finite Automaton (DFA) and parser handle the majority of WLN rules officially endorsed in the three major WLN manuals, with the parser showing a clear jump in accuracy and chemical coverage over previous submissions. The GitHub repository can be found here: https://github.com/Mblakey/wiswesser.

**Supplementary Information:**

The online version contains supplementary material available at 10.1186/s13321-024-00831-2.

## Background

In the field of computational chemistry, a key requirement is to represent molecular structures in machine readable formats [1]. These include file types that correspond to the complete molecular graph, such as SDF/Mol formats, and simpler string sequences known as chemical line notations. Whilst the majority of line notations generally do not include supplementary data beyond the traditional connection table [2], they are significantly leaner in size, and are a convenient method for compound sharing in large databases [3]. Regardless of the input format, chemical tool kits such as RDKit, OpenBabel and CDK [4–6] facilitate reading of the input format and construct an internal molecular object where various manipulations and transformations can take place. Due to these software packages, it is possible to interchange between formats depending on which is more well-suited to a particular objective[7]. Even traditional IUPAC nomenclature can be successfully parsed and converted by OPSIN [8]. Software such as NextMove’s LeadMine employs a grammar-based approach for the efficient parsing and extraction of chemical entities from documents [9, 10]. These grammars require expert construction, and offer highly effective matching of entities within the text. When coupled with format conversion capabilities, this entity matching process enables rapid extraction and curation of data, valuable for various cheminformatics tasks.

There are well-established algorithms for parsing and converting between line notations such as SMILES and InChI, which are contained in most conversion tool-kits [11, 12]. However, when it comes to notations predating these modern standards, there are either no existing processes, or concerns about their completeness and reliability [13]. As a result, access to information encoded in these older notations has been effectively limited to individuals with first hand knowledge of the notation’s rules.

Wiswesser Line Notation (WLN) [14] is one such line notation that predated SMILES, and saw widespread use amongst chemists and early cheminformatics software during its active years. Early look up systems such as the Index Chemicus Registry System (ICRS) [15] and the CROSSBOW software package [16] gave access to large chemical queries, and algorithms using WLN allowed for early substructure searching [17]. While the notation adheres to strict mathematical principles, the WLN language is conveyed through a set of verbose written English rules. As such, chemists had to be trained specifically in the rule set, and the input of compounds was done by hand on punch card machines [18], which was one of many reasons the notation was dropped in favour of systems with more rigorous foundations.

Due to the extensive distribution of chemical entities in WLN, and the importance for text mining and modernisation of old data [19, 20], there has been an interest in both recognising and parsing WLN, and creating the algorithms necessary for converting compounds represented in WLN to the modern formats. The first instance of a modern WLN reader was submitted to OpenBabel by Roger Sayle, claiming a 70% accuracy rate on the WLN strings available in the PubChem database [13]. As we will demonstrate in this work, this was likely an underestimate due to inaccuracies in the WLN strings themselves. Following this, a demo WLN reader was hosted by ChemDoodle, with similar results [21]. Both readers provided comprehensive coverage of acyclic compounds, but there was limited implementation of the rules for cyclic compounds. The encoding manuals that distributed WLN versions contained some revisions that omitted the cyclic rules, likely accounting for these gaps in coverage [22].

These parsers can attempt a conversion on a candidate WLN string, however when mining text, there is currently no method for extracting these candidate strings. In this work, we present a grammar for the WLN syntax, which subsequently enables fast identification and extraction of potential WLN strings, alongside a complete parser that covers all the WLN rules.

## Glossary

This section introduces some technical terms that will be used throughout the rest of the paper to describe the theoretical underpinnings of the software development.

- **Parsing:** The process of analyzing a sequence of symbols (usually text) to determine its grammatical structure or syntax.
- **Context-free grammar (CFG):** A formal language model used to describe the syntax of languages. It consists of a set of rules that specify how symbols can be combined to form valid strings.
- **Deterministic finite automaton (DFA):** A theoretical model of computation used to recognise regular languages. It processes input symbols one at a time, transitioning between states based on the input.
- **Non-deterministic finite automaton (NFA):** Another theoretical model of computation used to recognise regular languages. Unlike DFAs, NFAs can have multiple possible transitions for a given input symbol.

## WLN syntax and productions

The WLN rules are given in verbose English statements, with the most modern revision being seen in the encoding manual by Elbert G. Smith [23]. Whilst the notation was designed to have an unambiguous string for any given molecule, the design of the rules does leave room for interpretation on certain compounds, especially when it comes to aromaticity and kekulization in compound cycles. There are also additional constraints that are imposed by the WLN rules that limit the types of compounds that can be effectively represented. These limitations are inherent to how WLN represents rings, although possessing a valid representation, certain compounds would be impractical when expressed as WLN strings.

In order to parse WLN strings, the syntax and semantic structure needs to be represented in a formal way. One method is the Context Free Grammar (CFG), which uses a finite set of productions which encapsulate a language. A production is a simple rule or instruction that defines how to construct sentences or strings by replacing symbols with other symbols, enabling the formal representation of language structure. In this section, we give an base overview of the WLN rules, and how these can be formally represented as a set of productions for string parsing.

### WLN tokens and symbol definitions

**Fig. 1:**
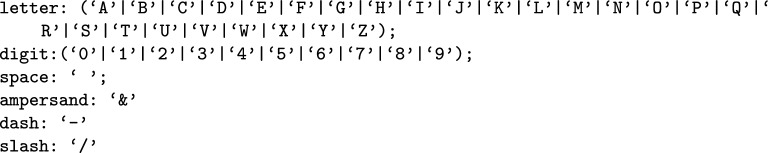
Terminal Characters (tokens) given for the WLN language

WLN is an encoding language consisting of strings of alphanumeric symbols, classified as a linear notation system. Formed of the alphabetic symbols [A-Z], punctuation symbols [-, &,/], numerals [0–9] and the space as a separator (Fig. 1). Historically the notation preceded the invention of ASCII [24], with only the upper case alphabetic characters available on the punch card computers of that era.

As the character set is so limited, each token naturally takes on various semantics depending on the syntax of the surrounding string. WLN syntax can be classified and separated into two primary environments: **Acyclic**, and **Cyclic**.

In the context of acyclic notation, tokens within WLN correspond to chemical elements or convey information about various levels of branching within the molecular structure. However, when situated within a cyclic environment, these symbols play a dual role. They not only define the presence of a ring but also serve as indicators of positions for acyclic substituents. These position indicators are commonly referred to as *locants*. Acyclic symbol definitions for the whole character set are shown in Table 1.

**Table 1:** WLN symbol definitions and their corresponding properties

WLN symbol	Meaning	Allowed branches	Terminating	Locant
A	Locant Only	0		$\checkmark$
B	Boron	3		$\checkmark$
C	Carbon	4 exactly		$\checkmark$
D	Open Chelate	0		$\checkmark$
E	Bromine	1	$\checkmark$	$\checkmark$
F	Fluorine	1	$\checkmark$	$\checkmark$
G	Chlorine	1	$\checkmark$	$\checkmark$
H	Hydrogen	1	$\checkmark$	$\checkmark$
I	Iodine	1	$\checkmark$	$\checkmark$
J	Ring Closure	0		$\checkmark$
K	Nitrogen (1+ charge, with 4 implied methyls)	4		$\checkmark$
L	Open Carbocycle	0		$\checkmark$
M	Secondary Amine (NH)	2		$\checkmark$
N	Nitrogen	3		$\checkmark$
O	Oxygen	2		$\checkmark$
P	Phosphorous	3		$\checkmark$
Q	Hydroxyl	1	$\checkmark$	$\checkmark$
R	Benzene	1 (expandable)		$\checkmark$
S	Sulphur	3		$\checkmark$
T	Open Heterocycle	0		$\checkmark$
U	Unsaturate bond	0		$\checkmark$
V	Carbonyl	2		$\checkmark$
W	Add -oxylate	0		$\checkmark$
X	Carbon (4 implied methyls)	4		
Y	Carbon (3 implied methyls)	3		
Z	Primary Amine (NH2)	0	$\checkmark$	
0..9	Alkane chain of length n	0		
** &**	Punctuation			
**-**	Punctuation			
**Space**	Punctuation			
**/**	Punctuation			

### Acyclic Productions

In instances where WLN is applied outside of a ring system, the symbols employed in the notation construct the molecular representation in a tree-like structure. Punctuation marks serve the purpose of specifying branches within the structure and demarcating ionic components. In this context, each alphanumeric symbol within WLN corresponds to either a chemical element or a frequently encountered functional group, where each symbol can only have one parent in the hierarchical tree-like structure. As a result of the condensation of functional groups, particularly in acyclic notations, WLN proves to be remarkably compact.

**Fig. 2:**
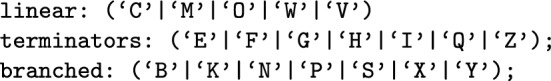
Degree specific productions given for the WLN language

Each functional symbol is then assigned a branching property that both enforces the allowed chemistry of an element, and removes the amount of punctuation needed in the string. These can be separated into distinct token classes, shown in Fig. 2.

**WLN degree**: Similar to SMILES, WLN strings represent the underlying molecular graph, allowing the assignment of a *valence* to each symbol. WLN bases its valence values on the number of allowed branches, rather than the sum of bond orders more commonly associated with atom valence. For instance, the symbol **Y** represents a carbon that can have three connected children, independent of whether one of these children is double bonded.

**Branching symbol**: Compound branches in WLN can only be started with specific characters, in general these have to be elements with a WLN degree greater than 2. All periodic code denotations are allowed to start branches, including single character hypervalence.

**Terminating symbol**: In the WLN notation, when a branch is initiated, certain characters are designated as terminating symbols. These symbols typically have a general valence of 1 or represent a group where the implicit hydrogen count does not permit further atomic definitions. These terminating symbols effectively close the branch, and the notation subsequently continues from the last branching symbol used.

#### Linear compounds

**Fig. 3:**
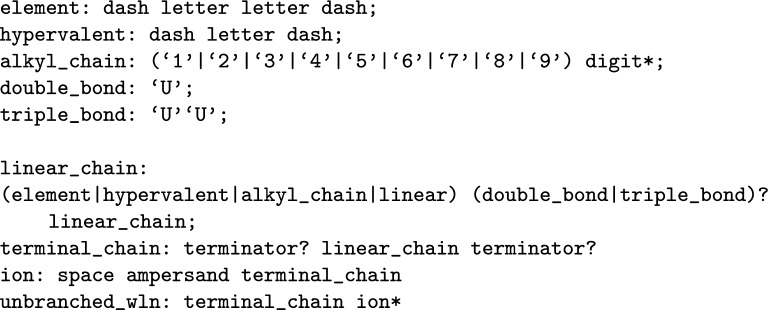
Linear compound productions given for the WLN language

**Fig. 4:**
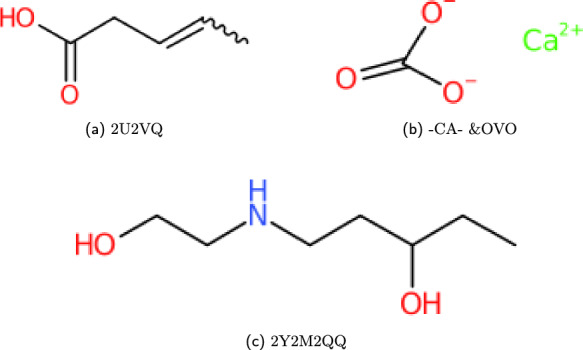
Smith reference compound examples created from the set of linear productions; bonding, ions and terminator characters are shown. Double bond stereochemistry has to be implied, as explicit descriptions were an notational element added in later versions

To define a linear compound, symbols can follow sequentially without any punctuation. If an element is required for which a specific symbol is not provided, the periodic character code can be inserted between two *-* characters. Alkyl chains are given as numerical strings, where the number indicates the number of carbons in the chain. It is important to note that in this context, if a terminator symbol is utilised anywhere except as the starting character, it promptly terminates the chain, preventing any further symbols from following it, except in cases where a new ion needs to be specified. To define an ion, the commencement of a new molecule can be signalled by a space followed by the ** &** character. For any bond unsaturations, the character **U** indicates the degree of the unsaturation (at most 2). These rules are represented as productions in Fig. 3 with example compounds given in Fig. 4.

It is important to recognise the recursive nature inherent in the *linear chain* production. This recursion is employed to ensure that the unsaturation character must have both a parent and a child to be considered valid within the context of WLN. While recursive rules such as this are the most straightforward means of representing WLN, it is crucial to acknowledge that, when it comes to creating a parser, enforcing such rules can pose challenges.

#### Branching compounds

**Fig. 5:**
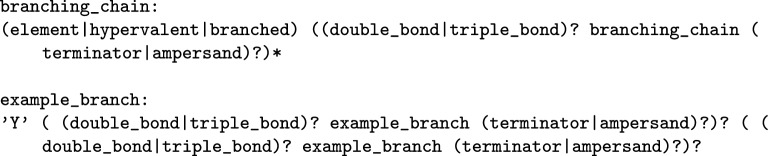
Branching compound production structure given for the WLN language. For specific branch counts, *example_branch* gives a structure that can be repeated for each available branch symbol

**Fig. 6:**
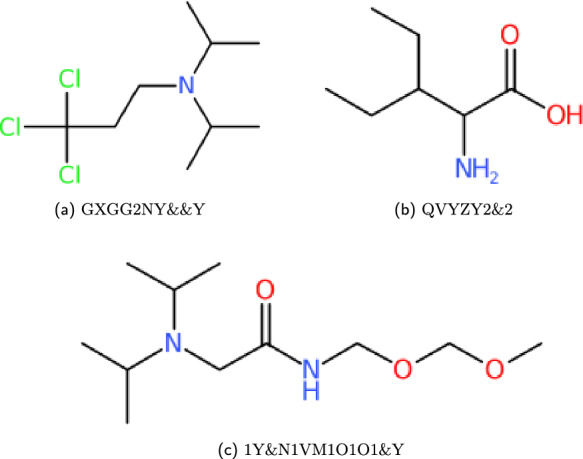
Smith reference compound examples created from the set of branching productions; The ampersand here is introduced here as a method for closing, and returning to previous branching characters

Within WLN, various symbols have the capacity to accommodate more than a single child, resulting in the formation of branching structures that can be interpreted through unbounded trees of WLN characters. In this context, a branch can be terminated through two distinct methods: either by employing an ** &** symbol or by utilising a terminator symbol. Upon the termination of a branch, the subsequent symbols become *attached* to the last branching character encountered. It is important to highlight that the presence of consecutive ** &** characters will lead to *popping back* (returning to previous) the designated number of branching symbols. When a branch is closed in this manner, no additional groups can be appended to that particular symbol. The productions for branching WLN strings replace the *linear_chain* production seen in Fig. 3, adding in a recursive rule to allow branching. The base structure of this production is shown in Fig. 5. In the *example_branch* production, the number of branches can be enforced with multiple recursive components. In this example, the symbol **Y** can have two subsequent branches, which is shown by two optional calls of the *example_branch* production on the right hand side. This structure can be followed for the other branching symbols, effectively limiting the branch count. Some examples are given in Fig. 6.

### Cyclic productions

The syntax for cyclic structures in WLN consists of two primary components: the cycle definition and any substituent groups intended to be attached to the ring. The cycle definition is encapsulated by the use of specific characters, namely (**L,T,D**) to initiate and (**J**) to close the cycle. Within this boundary, the notation describes the Smallest Subset of Ring Systems (SSRS) as well as any heteroatom designations and aromaticity assignments associated with the cycles within the subset. Heteroatom and R-group positions are defined by index lookup on a defined ring path using character locants.

#### The locant path

**Fig. 7:**
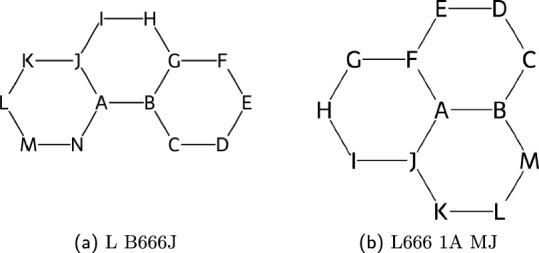
Locant Paths shown on chemical structures for** a** Phenanthrene and** b** Phenalene. WLN alphabetic indexing is shown on each atom. For Phenalene, the highest priority atom is the perifused center, and therefore it takes the starting point. Any further peri-fused points will force the path to travel in that direction

This ring path, called the locant path in WLN rules, is a singular path starting from a base atom indexed **A**, which traverses each atom only once until a loop is formed, see Fig. 7. Notably, the locant path concept in WLN is analogous to the Hamiltonian path concept in non-directed graphs [25]. It is important to recognise that there exists several valid cycle structures that do not possess such a path. For these compounds, a *ring branching* notation becomes necessary.

In the WLN rules, the starting position and direction of the locant path is given by the minimisation of a *locant sum*, defined as the sum of locant values shared between two sub-cycles. Nevertheless, it is often more intuitive to conceptualise this in terms of shared rings and allowed directions, which in turn give rise to the following foundational rules:

1. The starting position for the locant path is determined by the number of shared rings, the atom with the highest share count is the starting point. If the share count is above two, the atom is deemed *multicyclic*.
2. The locant path can never cross a fuse junction, unless to move to a multicyclic point.
3. If on a multicyclic point, and there is another multicyclic point connected, the path must travel through this second point.

#### Cycle structure

**Fig. 8:**
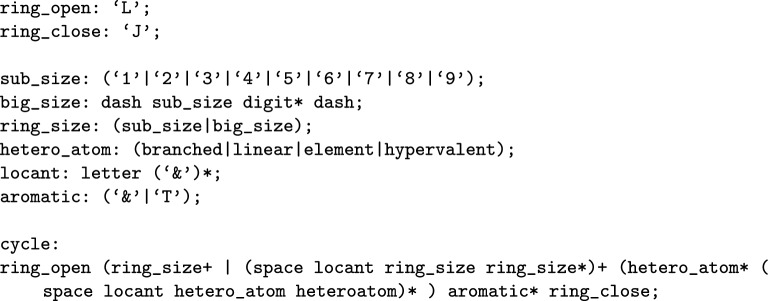
Basic cycle production structure given for the WLN language

**Fig. 9:**
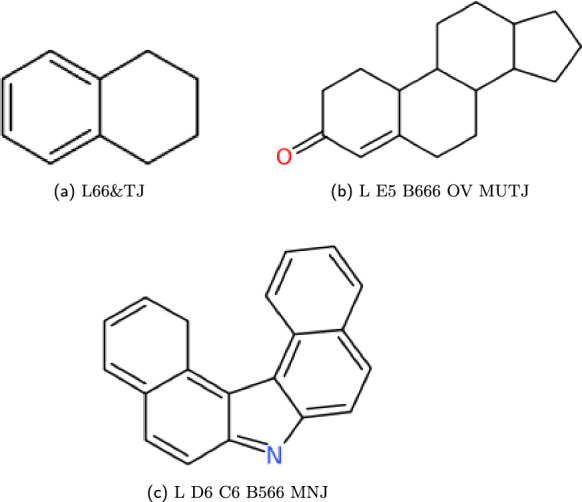
Smith reference compound examples created from the set of polycyclic productions; A demonstration is given for cycle open and closure, alongside heteroatoms and aromaticity assignments

As previously mentioned, the characters (**L,T,D**) initiate a cycle and the (**J**) character closes the cycle. Sub-cycle sizes are then given by their digit value, if the cycle size has more than one digit, they are wrapped between two **-** symbols, similar to how elements are defined outside of rings. Attachment position of the ring is either given with no locant symbol, which implies the ring is created around the **A** position in the path, or, a space followed by a locant indicates its lowest position. A locant symbol is any letter character up to **X**, to index past this, ** &** symbols are used. For example, in Fig. 7, Anthracene would be **L C666J**. Heteroatoms then follow with a similar format, although instead of taking on **A** if no locant is specified, the implied position is the position after the last locant seen. Prior to the closure of a ring within WLN, aromaticity designations are provided for each sub-cycle. Specifically, the symbols ** &** and **T** are used to signify aromatic or alphatic status, respectively. When only one of these symbols is provided for a sub-cycle, it applies to the entire system, indicating whether the entire system is either aromatic or aliphatic. These productions are given in Fig. 8 with example compounds seen in Fig. 9.

**Fig. 10:**
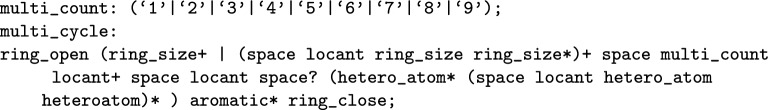
Multicyclic production structure given for the WLN language

**Fig. 11:**
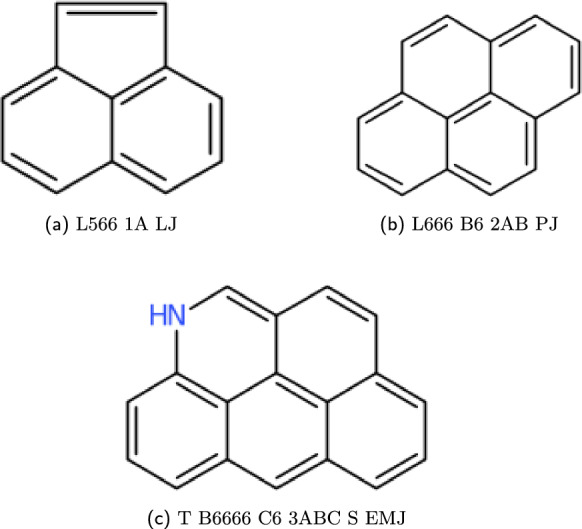
Smith Reference compound examples created from the set of multicyclic productions; Here the syntax expects a multicyclic block corresponding to the number of atoms shared between more then two subcycles

The production in Fig. 8 describes all ring systems that have atoms only sharing two or less sub-cycles. When this isn’t the case, further notation is needed which designates: the number and position of the multicyclic points and the size of the ring system as a locant value. These are both given before the heteroatom syntax, and syntax rules are shown in Fig. 10 with compound examples given in Fig. 11.

#### Bridges, crossed bonds and macro cycles

Specific rules are established for bridging positions and crossed bonds across a cycle, enabling the definition of complex interconnected ring structures. Bridges are represented using single-letter locants, separated by spaces, indicating the locant position that transitions into a bridging environment. Crossed bond notation is presented with a ring size followed by the character **/** and two locant characters, specifying the locants where the crossing of the bond begins and ends. Crossed bond notation offers a straightforward method for defining the bonds involved in ring junctions. However in the WLN rules, crossed bonds are primarily employed when no other means of representation are available.

**Fig. 12:**
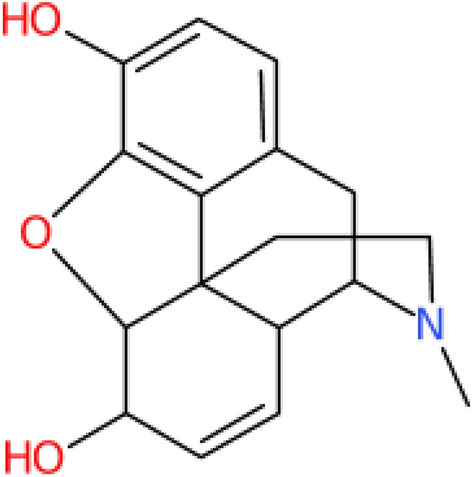
Morphine WLN: **T-T665 B6 2AB O KO NUT &TTJ IQ MQ B2N1 &- D6J**. Shown with macro-cyclic notation. Since Morphine has a valid locant path, this notation is left to the discretion of the user, an alternate form is **T B65 H6 F6 F6 3FGH R AO DU GX PN HU- MTT &TTJ CQ JQ P1**

**Fig. 13:**
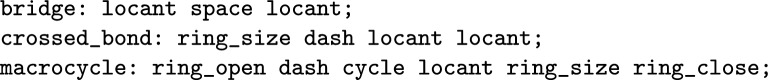
Bridge, crossed bond and macrocyclic productions for the WLN language

Macrocyclic compounds use an embedded style ring notation, which allows the definition of a branch that will wrap back to a specific locant position on the ring, demonstrated for the WLN string of Morphine, shown in Fig. 12. This does however have its limitations, which are apparent from looking at the last production in Fig. 13. The notation requires that before closing, the cycle defined in the embedded environment must have its sub-ring size defined. A consequence is that only one returning substructure can be added to any given ring. Productions for bridges, crossed bonds and macrocycles are given in Fig. 13. With bridges and crossed bond productions added as optionals into the general cycle rule. Discussions on this syntax revealed it was rarely used, as macro-cycles were typically outside the scope of WLNs usable chemical space, a comment not untrue to many modern line notations.

#### Locant substituents and spiro rings

**Fig. 14:**
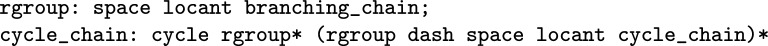
Recursive cycle chain productions for the WLN language

Once a ring system is closed, locants are then used to specify acyclic branches as R-groups. For chaining rings together, branches can be followed by **-** symbols and the attachment position of the new ring. Similar to regular branches, the ** &** symbol can be used to close and *pop back* rings, in order attach locants to a ring defined earlier. The productions for this again can be defined recursively, as technically an infinite chain of rings is allowed. Productions are given in Fig. 14;

The rules for a spiro compound are relatively simple, the locant branch character is substituted for an ** &**, and a chained ring is defined as previously stated, with the positional locant being the shared atom between the two rings.

## Matching WLN strings

For a given grammar, various parsing techniques are available to match a given input string. In the case of WLN, similar to IUPAC nomenclature, the grammar describes an infinite number of terms [26]. If parsing in a traditional way with a framework such as ANTLR, various techniques are used to handle the recursion but will come at the cost of run time speed, and are therefore not practical for text mining [27, 28].

Alternatively, one could employ a Finite State Machine (FSM) for parsing, which offers the advantage of linear time complexity [29]. We can also guarantee that the Deterministic Finite Automata (DFA) is minimal. However, for a DFA representation to be feasible, the language itself must be regular in definition.

### WLN text matching

To employ a DFA for matching WLN, certain rule relaxations are necessary. These relaxations result in strings that are syntactically valid according to the WLN rules but may not necessarily be semantically correct. The key modifications involve permitting any number of branches after a given symbol and allowing any number of closures at any point in the string. To address the omissions by relaxing the rules, a push-down style automaton with a stack can track open and closures of given branches. This method was heavily inspired by the bracket matching seen in LeadMine and CaffeineFix [26].

**Fig. 15:**
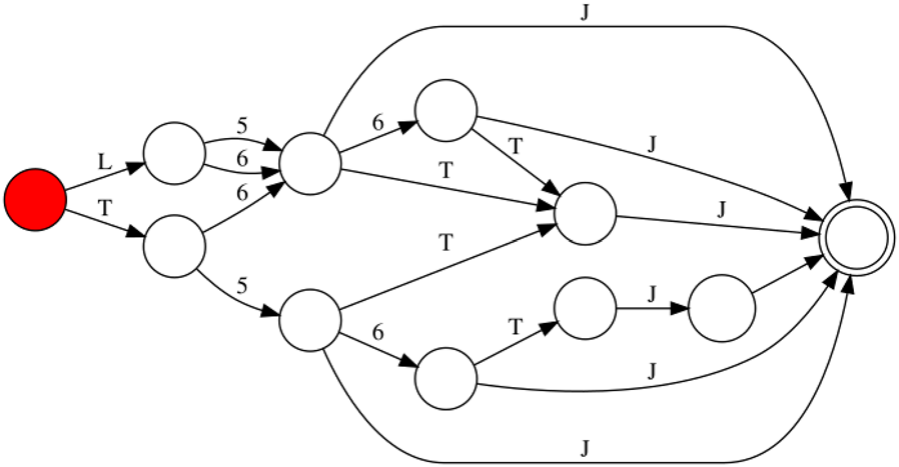
DFA that matches WLN strings for Benzene, Cyclopenta-1,3-diene, Cyclohexane, Cyclopentane,4 H-indene and Hexahydroindan. Root state is given in red, with the only accept shown with a double circle

DFA-based text matching offers a highly efficient method for extracting strings [30] that *could be* valid WLN expressions. Subsequently, these potential WLN strings can be further validated by the converter to ensure semantic correctness. An example FSM that matches simple 5 and 6-membered (bi)cyclic rings is given in Fig. 15.

In the process of constructing a DFA from the WLN rules, there exists greater flexibility in initially creating a Non-deterministic Finite Automaton (NFA) and subsequently converting it to a DFA through the subset construction method, followed by minimisation with the equivalence principle [29]. This approach results in a Finite State Machine (FSM) with a total of 59 states and 1028 edges, a small machine that enables very fast parsing.

**Fig. 16:**
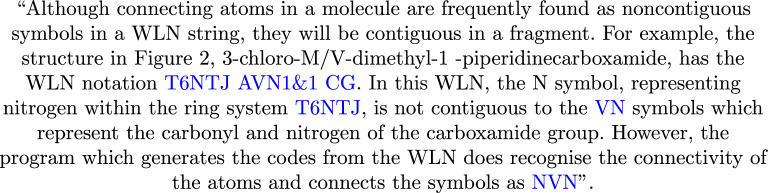
Excerpt taken from “Using the Wiswesser line notation (WLN) for online, interactive searching of chemical structures” [31], extracted WLN strings from *wlngrep* are highlighted in blue

The tool developed around this DFA, known as *wlngrep*, employs a greedy matching approach to extract WLN strings from text data. Similar to the conventions observed in the standard *grep* tool, matches can be retrieved using *wlngrep*. Exact matching will yield a result only if an entire line corresponds to a syntactically valid WLN string. If an entire line is not a valid WLN string, it will return the longest sequences of valid WLN strings present within that line. A consideration, is that most lone capital letters are themselves WLN strings, which would return matches for the start of any properly formatted sentence, or name/place. Therefore, single letter matches are turned off by default, but can be enabled with a flag if required. An example use is seen in Fig. 16.

## Parsing and converting WLN

**Fig. 17:**
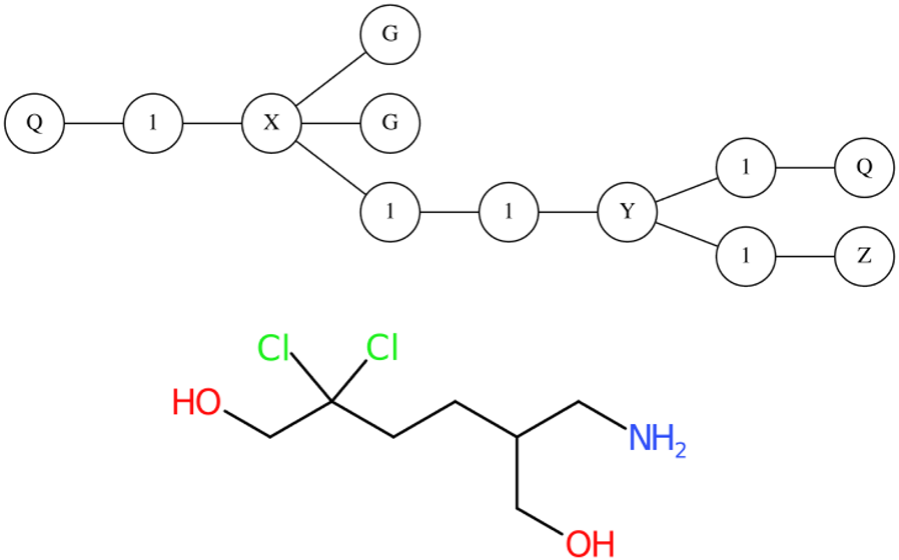
Interpreted WLN graph from input string: **Q1XGG2Y1Q1Z** and corresponding chemical graph from conversion to SMILES: **OCC(Cl)(Cl)CCC(CO)CN**. Numerical characters such as ‘2’ are turned into singular ones at graph formation. Other symbols such as **Q** and **Z** are evaluated at connection table creation. Note this WLN string does not follow Rule 2 canonicalisation rules

The base idea for parsing/reading WLN is to form a tree or directed component graph of the WLN characters for a given input string, see Fig. 17. This data structure captures all branching and cyclic features, which can then be transformed into the internal molecular graphs of any desired toolkit. Similar to the text matching in the DFA, the WLN string is read character by character, updating the WLN graph at each step. For cyclic structures, all ring information is needed before the cycle can be created, as such, the starting position of any open and close characters are recorded enabling the string to be spliced into separate routines. WLN symbols are often shorthand for functional groups, so these symbols need to be expanded after the graph is created.

For acyclic compounds building the graph is trivial, each character corresponds to a node in the graph, and can be built as the characters are read. For cyclic molecules, specific algorithms were developed to handle the different types of fuses and ring arrangements, whilst also maintaining the correct locant path. WLN has a strange way of denoting aromaticity, Hückel’s rule [32] does not need to be obeyed, instead a cycle is deemed aromatic if it has the maximum number of double bonds placed after heteroatom assignment. This is equivalent to the maximal matching problem in combinatorics, and solved easily for bipartite graphs using Ford-Fulkerson, and in the general case with Edmond’s Blossom [33]. Modern toolkits do employ this algorithm for kekulisation, but they tend to enforce stricter criteria regarding what is considered aromatic, in contrast to WLN, which has a more relaxed definition. Additionally, a deliberate design choice in the parser was to keep the majority of functions native, ensuring portability across different toolkits. It should be noted in later versions of the WLN rule set the aromaticity criteria was stricter, and at the discretion of the user, however for backwards compatibility across various WLN versions we maintain the relaxed scheme.

With the proper WLN graph constructed, symbols and edges can be read and interpreted as a SCT XI connection table, containing all atom types and bonds in an adjacency matrix. An advantage of this method is the WLN reader’s functions are all native, with a toolkit being able to simply loop through the atoms and edges to construct the internal molecular graph. The parser presented here uses OpenBabel to provide the subsequent conversions to SMILES or InChI, however with very few changes to the underlying source code this can work with RDKit or CDK.

### Rules supported

The following list is the WLN rule headings outlined in the revised version of Elbert G. Smith’s encoding book. All numbered rules given in the headings below are supported and handled by the parser and the reader. In addition to the standard set, all elements in the current periodic table (as of 2023) are supported by their 2 letter codes.

1. Unbranched and Branched Chains
2. Systematic Contractions
3. Organic Salts
4. Benzene Derivatives
5. Multisubstituted Benzene Rings
6. Benzene Rings in Branching Chains
7. Monocyclic Rings
8. Bicyclic Rings
9. Polycyclic Rings
10. Perifused Rings
11. Chains of Rings other than Benzene
12. Spiro Rings
13. Bicyclic Bridged Rings
14. Rings with Pseudo Bridges
15. Ring Structures with Crossed Bonds and Unbranched Bridges
16. Rings of Rings Contraction
17. Metallocenes and Catanenes
18. Chelete Compounds
19. Ionic Charges, Free Radicals and Isotopes

### Unsupported rules

The parser incorporates support for all fundamental rules, while certain provisional rules, such as mixture codes, inorganic formula notation, and stereoisomerism additions, have been intentionally omitted from its functionality. Additionally, the decision was made to adopt a more flexible approach with regard to the canonicalisation of WLN, deviating from the stringent character ordering stipulated by the official WLN specifications.

#### MANTRAP rules

Within the WLN, there exists an unpublished subset of rules. Readers learning the manuals will notice a gap in the rule set, eluding to a large set of rules that later revisions deemed unnecessary. These omitted rules likely constitute a subset of experimental regulations denoted by the acronym MANTRAP, representing Mixtures, Alternates, Not assigned, Tautomers, Reactants, Addition compounds, and Polymers.

In the context of more modern line notations, character representation of these areas of chemistry are still under development, with Tautomers being a large focus for the next iteration of InChI. Since these rules are undocumented in the official manuals, it was deemed sensible to leave them unsupported in the parser, rather then to guess what the rules might of been based on very limited examples.

#### WLN canonicalisation - rule 2

WLN did aim to achieve a unique canonical representation for each compound, where rules were created to enforce the ordering of symbols in various contexts, these include alphanumeric ordering, locant ordering in cycles, R-group ordering to name a few.

For example, outside of any cycle, preference is given to higher alphabetic characters. Figure 17 is a good example, the true canonical WLN for this compound would prefer the **Z** symbol to come before the starting **Q**, leading to **Z1Y1Q2X1QGG** being the technically correct string (Rule 2). Both examples are presented here to highlight a specific design decision made for the parser. The intention behind this software is to facilitate the modernisation of chemical documents. In this context, it cannot be assumed that all WLN strings were precisely encoded with the canonical rule set. It would be unreasonable to reject a string outright and not attempt a conversion if this were the case. Therefore, in the parser, canonical rules are not enforced.

### Implied WLN conventions

During the development of algorithms for WLN interpretation, it’s evident that the notation was originally intended for human interpretation, often by expert chemists who could infer implied information based on context. Hence, there exists a set of rules where validity hinges on users agreeing upon shared conventions. While these conventions may have been appropriate at the time, they introduce challenges when choosing to obey a convention or not in the parser design.

**Fig. 18:**
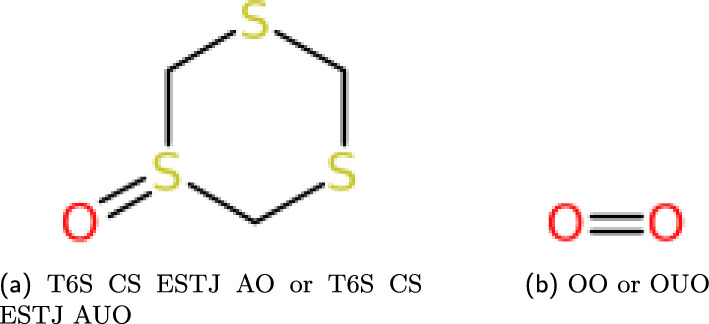
Smith Reference compound examples showing instances of implied double bonding. In both instances it would be valid to omit the **U** character

An example of this is implied double bonding, normally a bond unsaturation is given with a **U** symbol, however there are instances where the double bond could be assumed, and the **U** character omitted, an example is given in Fig. 18.

For an output SMILES, a valid implementation would be to balance charges by adding double bonds where necessary, however this would lead to some ionic species lacking a valid WLN representation. A simple example is the peroxide ion. In WLN, when two “O” characters appear together, it implies a double bond to form $O_2$, which is represented as **OO** or **OUO**. To represent hydrogen peroxide, you’d need to change the symbols to Hydrogen-attached Oxygens, resulting in **QQ**.

This poses a challenge in trying to represent the peroxide ion, $O_{2}^{2-}$. Conventionally, a negatively charged oxygen species is denoted with a single **O**.” As mentioned above, WLN automatically assumes the presence of a double bond in this context, even without using the **U** character, making **OO** unable to adopt a single bond representation. Tentative rules were later introduced to specify charges for each symbol, but these rules were considered supplementary and weren’t officially part of the language. For historical reasons, the WLN parser will default to implied double bonding, but a flag is available to turn this feature off, as this behaviour can lead to unexpected compounds.

### Error handling

Errors in the WLN processing can be identified and flagged in a couple of ways. Firstly, if nodes within the structure exceed their associated valence or if expected notations are absent during the parsing process, errors can be flagged immediately on the specific character and positions. Additionally, informative messages are provided to suggest potential changes that may resolve the issue.

For cyclic structures, it’s important to note that a single symbol may not always be the sole cause of an invalid WLN string. Therefore, when errors occur in cyclic structures, the closing **J** symbol is highlighted to pinpoint the problematic ring. Multiple error messages are then presented to assist the user in diagnosing and debugging the string effectively.

Some errors can be handled at run-time, for instance, certain WLN symbols correspond to the same element with different levels of WLN degree. If a connection exceeds the valence of a given symbol, in these cases, we have the option of *raising* the degree and changing the symbol to accommodate the potential increase. As an example, a common fault seen in the WLN strings is stating an **M** character for a branching nitrogen, where **M** disallows branching by definition. At run-time, we can raise this to an **N**, allowing the branching to continue as normal. Such errors are often encountered when working with older chemical data, where inaccuracies may arise during the OCR (Optical Character Recognition) process. Additionally, space characters are a known OCR-related fault, and measures have been implemented to skip space characters if they do not make semantic sense within a given WLN string.

## Conversion testing

**Fig. 19:**
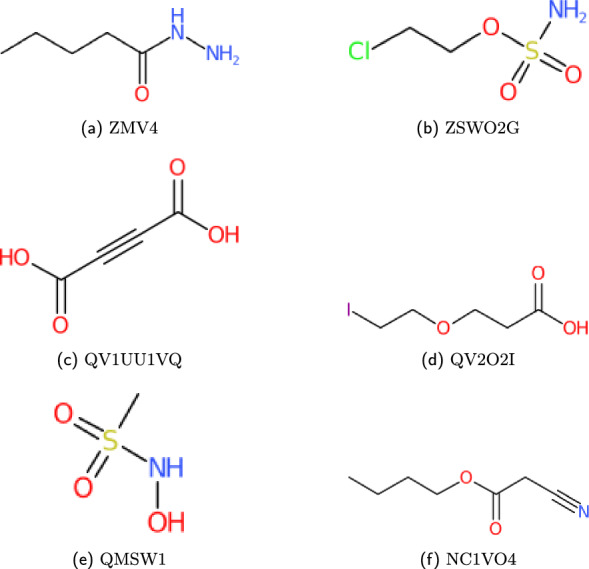
Examples of ChEMBL WLN compounds

**Fig. 20:**
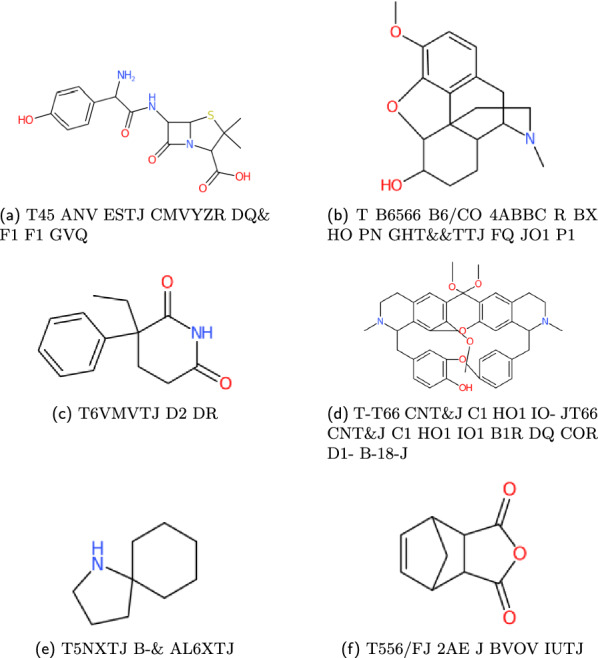
Examples of PubChem WLN compounds

**Fig. 21:**
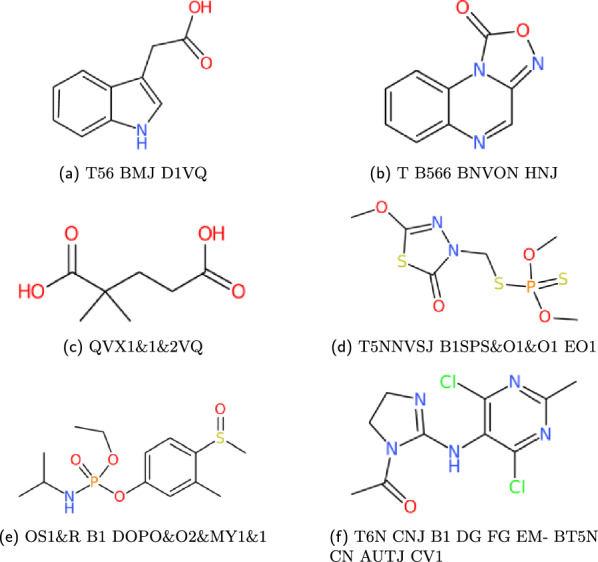
Examples of ChemSpider WLN compounds

For testing the precision of the WLN reader, the output SMILES must be compared against the identifiers given in external data sources. WLN identifiers with corresponding SMILES and InChI notations are accessible and searchable within databases such as ChEMBL [34], ChemSpider [35], and PubChem [36]. These databases provide public APIs that allow for simple retrieval. Compound structures are given in Figs. 19, 20 and 21.

A consistent method of testing, is to read in both molecules, output the canonical SMILES and do a simple string comparison between the two. This also ensures that all explicit/implicit hydrogens are correct, information that may be lost if comparing read in molecular graphs. For compounds that fail the conversion, it’s useful to assess how close the attempt was, which can be done by creating fingerprints for both the accepted SMILES in the database, and the output SMILES from the WLN reader, then subsequently computing the Tanimoto similarity coefficient. A score close to 1 indicates small changes needed to either the WLN string or the algorithms developed. Before fingerprint generation, it is also key to remove any stereochemistry from the public identifiers, as WLN inherently had little to no stereo information in the notation. Compounds represented with WLN typically lie in the small drug-like region of chemical space, and as such, an FP2 Fingerprint generated with OpenBabel is an appropriate choice for computing the similarity [37].

With an exception to the WLN strings given in Smith’s encoding book, the correctness of the WLN string also needs to be evaluated. A pipeline using the *wlngrep* tool is therefore set up:

- Attempt to match the string with *wlngrep*.
- If the whole string passes exactly, test the full WLN string conversion with its associated SMILES.
- If the string is partially correct, test extracted valid WLN sub-strings against the SMILES. Recording the incorrect string and potential corrections.
- Else the string is not a suitable test case, and flagged as an incorrect WLN.

### Benchmark rule set

Within Elbert G. Smith’s encoding reference book [23], each rule definition is accompanied by illustrative compounds presented as WLN strings along with corresponding structural formulas. Following the implementation of these rules, a comprehensive benchmark dataset consisting of 412 WLN compounds was curated across all the rules supported, giving a diverse set to assess precision. The development of the reader was grounded in these rules and examples, leading to an expected and indeed observed conversion precision of 100%. Naturally, as a WLN encoding book, all the WLN strings passed exact matching with *wlngrep*.

In this context, where SMILES representations were not provided, a evaluation of the similarity between the generated SMILES and the source compounds’ drawn structures was conducted to ensure correctness before associating the accurate SMILES with the respective WLN string. With the exception of selective rule procedures that were intentionally omitted, all WLN strings were successfully parsed.

### ChEMBL

In the ChEMBL database, there exists a collection of 2934 compounds with associated WLN identifiers. Using the DFA confirmed that nearly all 2934 WLN strings are syntactically valid under exact matching. The failed strings were traced back and all attributed to the WLN character **C** which is meant to force a full valence carbon with at least one unsaturated bond, either triple or double. Since this bonding is implied, use of the **U** symbol is both redundant and disallowed notation. For instance, the WLN string **SCN** corresponds to SMILES: SC#N, where the presence of a triple bond between the carbon and nitrogen atoms is implied. The ChEMBL entry for this compound is given as **SCUUN**.On conversion, only three compounds did not produce the expected SMILES, all attributed to these bond order descriptions. In this case, we can conclude our precision on read is at 100% with a correction required in ChEMBL.

### PubChem

For PubChem, 6589 compounds are associated with WLN identifiers. Exact matching reveals that only 5745 of these strings follow accepted WLN syntax. While it is not possible to list all the rejected strings here, a comprehensive list of the rejected strings will be provided for each relevant set. WLN strings sourced from PubChem have evidently undergone OCR, resulting in a considerable number of errors that are typical for OCR’d text. An example is the WLN string **Z2Z & GH**, where a spacing between the ** &** and the **G** is invalid notation. Other errors are due to WLN strings that whilst syntactically valid, could not possibly equal the SMILES they are associated with. During the conversion testing process on the remaining WLN strings from PubChem, accurate SMILES representations were obtained for 4934/5745 strings, which accounts for a success rate of slightly over 85%. A review of the WLN strings and their corresponding expected SMILES revealed a substantial number of incorrect WLN strings.

### ChemSpider

ChemSpider is the largest data source available, with 15,941 compounds present with corresponding WLN strings. Comparable to PubChem, not all strings pass on exact match, giving a total of 12,949 valid WLN candidates. The parser achieved an accuracy score of 11,962/12,949, and 92% as final precision on the syntactically correct WLN entries. Errors in the WLN strings are consistent with the findings seen in PubChem.

### Results summary

Table 2 summarises precision and matching success across all the data sources from the new parser. We also provide the precision seen from the old parser in OpenBabel. The conversion values given here are from the greedy matching procedure, with percentages relative to the starting set size instead of the exact matches given earlier. Greedy matches are given alongside exact matches, where all valid sub-strings are extracted. The greedy procedure can extract multiple sub-strings per line, accounting for a match value over the starting size for ChEMBL, PubChem and ChemSpider.

**Table 2:** Results summary of the new parser from the Smith, ChEMBL, PubChem and ChemSpider WLN conversion and match testing

Data set	Set size	Exact matches	Greedy matches	New parser	Old parser
Smith WLN	421	421	421	421 (100%)	217 (52%)
ChEMBL	2934	2931	2934	2931 (99.8%)	2930 (99.7%)
PubChem	6589	5745	7810	4934 (75%)	4364 (66%)
ChemSpider	15941	12949	20264	11962 (75%)	11526(71%)

Considering the old parser only contained rules up to bicyclic rings, the results on external data are much closer than first imagined. This suggests that the WLN strings present in the external data primarily adhere to the rules outlined in the initial chapters of the encoding books. Which, is somewhat expected as several renditions of encoding books stop before complex cyclic structures are discussed.

### Incorrect WLN entries

**Fig. 22:**
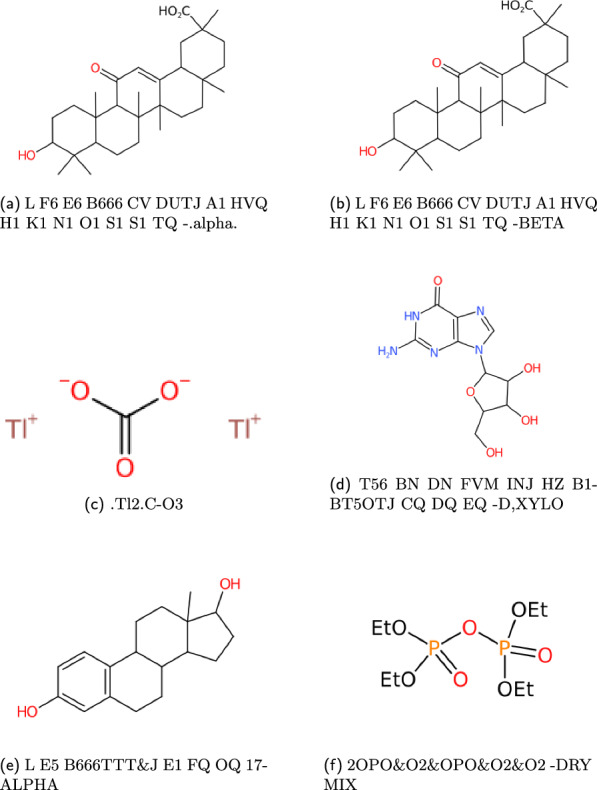
Examples of WLN entries that failed initial conversion. Strings (**a**,** b**) contain codes for specifying alternate forms, which should either be given after a double ** &&**. (**c**,** d**) are cases where disallowed characters have been mixed into the notation, specifically dots and commas. (**e**,** f**) contains MANTRAP specifiers, but incorrect appended

Within the databases PubChem and ChemSpider, there are roughly 10% WLN identifiers that may appear incorrect based solely on the rule criteria outlined in Elbert G. Smith’s revised edition [14]. Nonetheless, this observation does not imply that these supplementary string elements were inappropriate when initially employed. Historically, chemists might have used personalised abbreviations to depict structures that the WLN system struggled to comprehensively or conveniently represent within the confines of the accepted rule set. Examples of such strings are given in Fig. 22.

At a first glance, some of these could be omitted very easily, due to the use of lower case characters which were never specified in the language. This however is not the right approach, discussions with Barrie Walker gave some insight into string suffixes, where a double ampersand (** &&**) followed by any valid description would still yield a valid WLN string. These suffixes could be a mixture of upper or lower case and were commonly used in the Commercially Available Organic Chemicals Index (CAOCI), a historic data repository that contained available compounds with both IUPAC and WLN strings [38]. For example, in Fig. 22a, a valid correction would be **L F6 E6 B666 CV DUTJ A1 HVQ H1 K1 N1 O1 S1 S1 TQ &&Alpha**.

Chemspider contains a lot of these string additions (submitted by Barrie Walker himself), and are instances of completely valid notation. A quantitative test is to use *wlngrep* to remove the suffix, and parse test the remaining string with the target compound, in all cases this produced a successful match.

String additions without the double ampersand are more problematic, as upper case codes are a common format for the MANTRAP rules, as mentioned in Sect. MANTRAP Rules. In cases where the suffix is all uppercase, and the remaining WLN string successfully matches the target SMILES, it is reasonable to assume that this form assignment was valid, and no correction will be provided. In all other instances the suffix can be either omitted, or corrected to the double ampersand format and resubmitted to the corresponding repository.

## Conclusions and future work

In conclusion, tools for both the extraction and conversion of WLN from chemical documents have been successfully developed. Both the DFA and parser handle the majority of WLN rules officially endorsed in various WLN manuals.

The parser demonstrates strong performance across a wide range of compound types, from four different sources. Successfully parsing all the WLN strings provided in the rule book instils a high degree of confidence that the rules have been accurately and comprehensively described. Of particular note is the ability to effectively handle cyclic structures, representing a clear advancement compared to previous submissions. Both systems will enable the community to modernise legacy notation data that otherwise would have remained unusable.

Nonetheless, inaccuracies within the external data sources present a challenge when attempting to precisely quantify the true conversion accuracy. We can however confidently establish a lower bound of no less than 80%. It is worth emphasising that the development of these tools has led to the identification of errors in industry-standard data sources. In turn, enabling corrections and improvements that benefit the scientific community Additional files 1,2, 3 and 4.

Future work will encompass the development of methods for correcting these WLN strings, with the intention of formally submitting these corrections to the respective libraries. To facilitate this process, we will also submit a WLN writer to OpenBabel. The writer will allow for the transformation of input SMILES and InChI notations into WLN. At the time of publication, all rules have been successfully covered, and will be presented in future articles. We will also be exploring whether WLN itself has any merit as an intermediate for string based cheminformatics, requiring the round-trip and conversion of well-curated standardised data sets. We hope these tools help the modernisation of any legacy chemical documents.

### Supplementary Information

**Additional file 1.** smith.tsv (WLN:SMILES strings from Elbert Smiths encoding manual).

**Additional file 2.** chembl.tsv (WLN:SMILES strings extracted from ChEMBL).

**Additional file 3.** pubchem.tsv (WLN:SMILES extracted from PubChem).

**Additional file 4.** chemspider.tsv (WLN:SMILES extracted from ChemSpider).

## Data Availability

Code availability - This project was written in C++ and built with CMake and the GCC compiler, we hope this will be merged into OpenBabels main branch, however until then a development repository that builds OpenBabel externally is given in supplementary information. A forked branch of OpenBabel is also given for those wanting to use the python bindings in OpenBabel instead of the Command Line Interface (CLI) provided in our own repository. Link to GitHub repository is https://github.com/Mblakey/wiswesser. Four files have been submitted to supplementary information, these are.tsv files containing the WLN and SMILES from each data source discussed in this paper. For PubChem, ChemSpider and ChEMBL, these were obtained through their public APIs. We have also included a.tsv file from Elbert G. Smiths encoding book, where SMILES and corresponding WLN were corded ourselves.
